# Sarcopenia and Chronic Complications of Type 2 Diabetes Mellitus

**DOI:** 10.1900/RDS.2022.18.157

**Published:** 2022-09-30

**Authors:** Dyah Purnamasari, Erpryta Nurdia Tetrasiwi, Gracia Jovita Kartiko, Cindy Astrella, Khoirul Husam, Purwita Wijaya Laksmi

**Affiliations:** 1 Division of Endocrinology Metabolism and Diabetes, Department of Internal Medicine, Dr. Cipto Mangunkusumo National Referral Hospital, Faculty of Medicine, Universitas Indonesia, Jakarta, Indonesia,; 2 Metabolic Disorder, Cardiovascular and Aging Research Center, The Indonesian Medical Education and Research Institute, Faculty of Medicine Universitas Indonesia, Jakarta, Indonesia,; 3 Department of Internal Medicine, Dr. Cipto Mangunkusumo National Referral Hospital, Faculty of Medicine, Universitas Indonesia, Jakarta, Indonesia,; 4 Division of Geriatric, Department of Internal Medicine, Dr. Cipto Mangunkusumo National Referral Hospital, Faculty of Medicine, Universitas Indonesia, Jakarta, Indonesia.

**Keywords:** type 2 diabetes mellitus, sarcopenia, atherosclerosis, neuropathy, nephropathy, retinopathy

## Abstract

Sarcopenia, defined as the loss of skeletal muscle mass and strength and/or a decrease in physical performance, is classically related to aging. However, chronic disease, including type 2 diabetes mellitus (T2DM), may accelerate the development of sarcopenia. Previous studies found strong association between T2DM and sarcopenia. Insulin resistance that exists in T2DM is thought to be the key mediator for impaired physical function and mobility which may lead to sarcopenia. T2DM may cause sarcopenia through the mediation of insulin resistance, inflammation, accumulation of advanced glycation end-products, and oxidative stress that may affect muscle mass and strength, protein metabolism, and vascular and mitochondrial dysfunction. On the other hand, loss of muscle in sarcopenia may play a role in the development of T2DM through the decreased production of myokines that play a role in glucose and fat metabolism. This review highlights the findings of existing literature on the relationship between T2DM and sarcopenia which emphasize the pathophysiology, chronic vascular complications, and the course of macrovascular and microvascular complications in T2DM..

## 1 Introduction

Diabetes mellitus (DM) is a chronic disease characterized by hyperglycemia, caused either by impaired insulin secretion or insulin function. More than 90% of DM is type 2 DM (T2DM) with an underlying mechanism of insulin resistance [1]. Worldwide, there are 537 million people with DM with highest prevalence among those aged 50-59 years [2]. In young T2DM patients, chronic microvascular and macrovascular complications occur earlier, during productive age [1].

The risk of macrovascular complications, such as myocardial infarction, increases 14-fold in the population diagnosed with T2DM before the age of 45 years [1]. In one study, macrovascular complications were significantly associated with mortality rate (HR = 2.00, 95% CI = 1.69-2.38) and microvascular complications such as nephropathy, neuropathy, and untreated retinopathy contributed to increased mortality [3].

The existence of insulin resistance is associated with the accumulation of abdominal visceral fat and obesity [1]. Accumulation of fat in muscle tissue will trigger proinflammatory cascade leading to mitochondrial dysfunction, impaired insulin signaling, and muscle wasting [4]. The reduction in muscle mass results in glucose storage disruption, resulting in further interference of glucose storage and insulin sensitivity [5]. Insulin resistance found in T2DM is an indirect risk factor for impaired physical function and mobility, as T2DM is associated with a 3-fold risk of sarcopenia and 8.2% of newly diagnosed T2DM also have sarcopenia [6].

The 2019 Asian Working Group for Sarcopenia (AWGS) defines sarcopenia as loss of skeletal muscle mass and strength and/or a decrease in physical performance [7]. Along with aging, decrease in muscle mass and strength can occur as body composition changes. In addition, fat infiltration into muscles contributes to decreased muscle strength [4]. Since sarcopenia is commonly related to aging, most studies of sarcopenia involve populations over 60 years of age [8]. However, sarcopenia also can occur at younger age. In a study conducted in Korea, researchers found that the prevalence of sarcopenia in adolescents aged 20 years was 15% and increased with age, with high blood pressure, increased triglycerides, obesity and T2DM as influencing factors [8]. Loss of muscle mass and strength can lead to limitation of mobility, increased risk of falls and fractures, as well as functional impairment and increased dependence [6].

Sarcopenia is associated with macrovascular and microvascular complications of T2DM. Several studies showed that low muscle mass was associated with increasing risk of atherosclerosis, prevalence of coronary calcification, and all-cause mortality and major adverse cardiovascular event through insulin resistance and inflammatory pathways [9,10]. Presence of retinopathy, nephropathy, and neuropathy in T2DM patients have shown to increase risk of developing sarcopenia, through reduced mobility in retinopathy and diabetic foot patients, and chronic inflammation and advanced glycation end-products (AGEs) in nephropathy [11-13].

Despite having strong association, the exact mechanism or whether it could affect both ways, still needs to be investigated further. Therefore, this review aims to summarize the relationship between T2DM and sarcopenia which emphasize on pathophysiology, chronic vascular complications, and how far sarcopenia affects the course of macrovascular and microvascular complications in T2DM.

### 1.1 Impact of diabetes on sarcopenia

In patients with T2DM, the incidence of sarcopenia is 2-3 times higher compared to patients without T2DM [14]. Trierweiler et al. [14] found that sarcopenia was diagnosed more in the T2DM group than in the control group (16.2% vs. 2.4%; p = .01). T2DM is associated with poorer skeletal muscle strength and quality, and a greater loss of muscle mass. Duration of T2DM of more than 6 years and HbA1c levels above 8% were associated with poorer muscle quality. Thigh muscle mass was found to decrease twice as fast in T2DM patients [6,15].

There are several mechanisms known contributing to the occurrence of sarcopenia, such as insulin resistance, inflammation, accumulation of AGEs, and oxidative stress, all of which can also affect muscle mass and strength, protein metabolism, vascular and mitochondrial dysfunction, and cause cellular death [5]. Other mechanism, such as peripheral neuropathy, is also thought to be involved in the development of sarcopenia in diabetes.

### 1.2 Insulin resistance

Insulin resistance is the hallmark of T2DM. Insulin resistance induced by impaired insulin signaling pathway disturbs translocation of glucose transporter (GLUT)-4 that is needed for glucose uptake primarily in striated muscles and adipose tissue. Insulin stimulates filamentous actin (F-actin) reorganization by binding of actin to myosin and phosphorylation of myosin. These will initiate myosin to assist GLUT-4-containing vesicle to colocalize along actin and to contract actin. Actomyosin cytoskeleton contraction results in membrane reorganization needed for fusion of GLUT-4 at plasma membrane. Thus, insulin resistance will impair glucose uptake in skeletal muscle and adipose tissue [16]. Not only interfering with glucose uptake, insulin signaling impairment also decreases glycogen synthase activity leading to reduced glycogen synthesis. Taken together, muscle loses its fuel, making protein degradation more favorable in times of energy demand [17].

Insulin resistance in patients with or without T2DM is associated with poor muscle quality. The decrease in muscle mass and strength in patients with poor glycemic control may be due to increased protein degradation and reduction in protein synthesis [18]. Insulin resistance in muscles can decrease anabolic response to exercise and amino acids. Moreover, increase in leptin resistance involved in adipocytokine dysregulation, contributes to ectopic fat deposition by reduced fatty acid oxidation in muscles causing myosteatosis. Fat accumulation in skeletal muscle (intramyocellular fat infiltration and intermuscular adipose tissue, IMAT) is associated with metabolic disturbance and inflammation through increased local proinflammatory cytokines and free fatty acids, which then activates macrophage and p38 mitogen-activated protein kinase (MAPK)-mediated insulin resistance. Mitochondrial dysfunction induced by intramuscular fat is caused by impaired β-oxidation and increased reactive oxygen species (ROS) formation, causing lipotoxicity and secretion of proinflammatory myokines, thereby resulting in atrophy of skeletal muscles [4]. Therefore, IMAT is highly predictive for both muscle and mobility functions in elderly.

### 1.3 Inflammation

In T2DM, the occurring chronic low-grade inflammation affects glucose and muscle homeostasis. Visceral adipose tissue produces proinflammatory interleukin (IL)-6 and tumor necrosis factor (TNF)-α which are inversely related to muscle strength, quality, and performance [5]. Lipid accumulation in adipose tissue related to obesity activates c-Jun N-terminal kinase (JNK) and nuclear factor kappa B (NFκB) signaling pathways that increase insulin resistance by impairing insulin signaling through serine kinase phosphorylation of insulin receptor substrate (IRS)-1 and by producing proinflammatory cytokines such as TNF-α, IL-6, and IL-1β, which also activate JNK and NFκB pathways all over again [19]. These activate a ubiquitin-proteasome system leading to protein degradation that results in muscle atrophy [4].

IL-6 is thought to increase muscle catabolism, whereas IL-10 is believed to act as an anti-inflammatory cytokine that suppress pro-inflammatory cytokines [20]. It turns out that IL-6 has both a proinflammatory and an anti-inflammatory nature. Proinflammatory IL-6 is produced by macrophages, whereas anti-inflammatory IL-6 is produced by striated muscles. When exercising, myocytes produce IL-6, which is useful for nutrient utilization and muscle hypertrophy [21]. Conversely, IL-6 also has been reported to cause muscle atrophy when administered locally to experimental animals [22]. Park et al. [23] found that patients with T2DM aged 70-79 years had a greater loss of leg muscle mass and strength and a decreased association strength after adjustment for IL-6 and TNF-α levels. Another study by Yu-Dong Rong [20] investigating IL-6 and IL-10 in 82 elderly Chinese population with sarcopenia found that IL-6 level and IL-6/IL-10 ratio were positively associated with sarcopenia.

IL-10 released by macrophages in adipose tissue improves insulin sensitivity by suppressing IL-6 and reversing TNF-α-mediated impaired insulin signaling. Its ability to suppress proinflammatory cytokines such as IL-6 and TNF-α also favors myogenesis by counteracting inflammation-induced muscle loss [24]. In obesity, the lower circulating level of IL-10 is associated with metabolic syndrome which then contributes to development of insulin resistance and muscle loss [24].

An overactive mechanistic target of rapamycin complex 1 (mTORC1) is thought to be the center part of many aging-related conditions including sarcopenia. In their study of Sprague-Dawley rats, Joseph et al. [25] found upregulated mTORC1 signaling up to 10-fold in rats aged 27 months with sarcopenia compared to those aged 6 months. Treatment with rapalog, a derivative of rapamycin, which inhibits mTORC1 signaling, showed improvement of muscle mass and morphology [25]. Although mTORC1 normally is needed in protein synthesis and muscle mass gain, it is suggested that overactive mTORC1 instead damages muscle fiber in sarcopenia. Chronically overactivated mTORC1 due to excessive glucose intake will perpetually lead ribosomal protein S6 kinase 1 (S6K1) to serine kinase phosphorylation of IRS-1, resulting in insulin resistance because of impaired insulin signaling. The activation of mTORC1 itself also can result in IRS-1 degradation leading to insulin resistance [26].

Regarding this phenomenon, hyperglycemia-induced chronic overactive mTOR1 might be connected with the mechanism proposed by this study, linking T2DM to the development of sarcopenia.

### 1.4 Oxidative stress and AGEs

T2DM is associated with an increase of oxidative stress which may lead to myopathy. This oxidative stress may occur through lipid metabolism disorders, insulin resistance, increase in AGEs, or mitochondrial dysfunction [5]. Zacarías-Flores et al. [27] found that an increase in oxidative stress was inversely related to skeletal muscle mass. Patients with T2DM had a recovery half-life of phosphocreatine, a measure of mitochondrial function after exercise that was 45% lower than that of the control. Moreover, first-degree relatives of T2DM patients had a 38% lower mitochondrial density than did control patients [5].

Hyperglycemia induces the overproduction of superoxide anions by several mechanisms. Glucose uptake increases NFκB binding in mononuclear cells, causing extensive muscle damage in experimental animals through increased expression of proteasome C2 and C9 subunits and MuRF1 genes [18]. Insulin itself decreases proteasome catalytic activity in muscles. In conditions of insulin resistance, high plasma glucose levels may lead to muscle atrophy. A murine experimental study by Russell et al. [18] reported that high glucose level caused muscle atrophy either through decrease in protein synthesis or increase in protein degradation. This mechanism is thought to occur via the proteolytic pathway of the ubiquitinproteasome preceded by activation of protein kinase R (PKR).

Non-enzymatic reactions of glucose, proteins, lipids, and nucleic acids can lead to the formation of AGEs. At present, there are no clear mechanisms by which AGEs cause muscle disorders. The proposed mechanism is an increase in cross-linking protein causing impaired muscle contractility, inflammation, and oxidative stress [5]. Skin autofluorescence, as a marker of AGEs, can reflect long-term glycemic control as well as lower muscle mass and strength [28]. Mori et al. [28] showed increased skin autofluorescence in T2DM patients with sarcopenia, compared to T2DM patients without sarcopenia.

## 2 Impact of sarcopenia on diabetes

Striated muscles are responsible for 80%-90% of glucose utilization, and also, production of myokines [5]. Loss of muscle leads to decreased production of myokines, which have been known to be beneficial in glucose and fat metabolism. Myokines are cytokines and peptides produced by muscles during physical activity that can reduce inflammation and prevent lipid accumulation and worsening of sarcopenia [29]. IL-6 produced by muscles is different from proinflammatory cytokines produced by macrophages and has several roles in maintaining glucose and lipid homeostasis. In response to muscle contraction during physical activity, IL-6 production will be increased especially in low muscle glycogen condition, activating AMP-activated protein kinase (AMPK) and phosphoinositide-3 (PI3) kinase to increase glucose uptake, increasing translocation of GLUT-4 to cell membrane to increase glucose uptake, and increasing hepatic glucose production [29].

**Figure 1.**
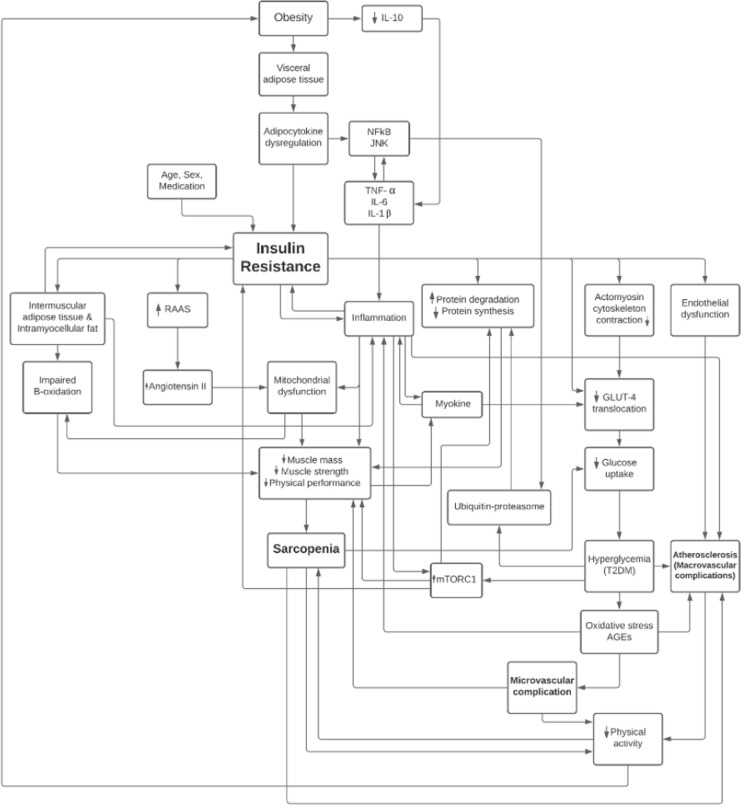
Summary of relationship between type 2 diabetes mellitus and sarcopenia.

A cohort study by Hong et al. [30] found that muscle mass was inversely associated with the incidence of T2DM in previously healthy individuals. This association was found to be stronger in the age group under 50 years, with a mean age of 38.5 years. A national study by Srikanthan et al. [31] found that muscle mass was associated with insulin sensitivity and a lower risk of prediabetes. For every 10% increase in skeletal muscle index, a decrease of 11% in HOMA-IR (95% CI 6-15) and a decrease of 12% in the incidence of prediabetes (95% CI 1-21%) was found [31] (Figure 1).

## 3 Relationship between sarcopenia and macrovascular complication of T2DM

### 3.1 Sarcopenia and atherosclerosis in patients with T2DM

T2DM leads to the development of macrovascular complications such as atherosclerosis, peripheral arterial disease (PAD) and stroke, with prevalences of 29.1%, 20%, and 7.6% respectively in T2DM adults. Atherosclerosis is key in the development of T2DM- related complications [32,33]. The poor metabolic state of T2DM is likely to contribute to its faster development in individuals with T2DM. Chronic hyperglycemia also attenuates vasodilation by increasing the accumulation of AGEs, oxidative stress, and direct actions on nitric oxide synthase on endothelia. These processes lead to hypertension, which in turn, leads to end organ damage [34]. Interestingly, an end organ that may be involved is skeletal muscle, as it is also part of the vascular territories affected by hypertension. Hypertension has been shown to cause *in vivo* damage to myocyte and increased peripheral vascular resistance that can be found in all vascular areas, including the skeletal muscle. In the presence of hypertension, T2DM, or obesity, significant degenerative remodeling can occur, resulting in the loss of arterioles and capillaries, a process known as vascular rarefaction [35]. In the renin angiotensin aldosterone system (RAAS), angiotensin II activity which is essential in blood pressure regulation, plays an important role in the development of insulin resistance and is fundamental to muscle regeneration and wasting [36]. Taken together, these data show that hypertension, which can be a complication of insulin resistance in T2DM, is a comorbidity that accelerates the development of atherosclerotic cardiovascular disease and sarcopenia.

In a study of 8.202 Korean adults with T2DM, Seo et. al. [9] reported that 52.4% of patients had carotid atherosclerosis. The prevalence of carotid atherosclerosis increased with decreasing skeletal muscle mass in both T2DM adult males and females. A shared pathophysiological pathway between low skeletal muscle mass and atherosclerosis is thought to explain this finding. Changes in myokine profile (such as increased levels of IL-6; TNF-α; c-reactive protein, CRP; and decreased levels of growth hormone, GH; and insulin-like growth factor-1, IGF-1) due to low skeletal muscle mass and aging can exert proinflammatory effects that may contribute to the progression of atherosclerosis [37]. Furthermore, decreased muscle mass can worsen insulin resistance, and hyperinsulinemia activates the Akt/IKK signaling pathway followed by activation of NFκB and subsequently the systemic inflammatory cascade. The negative correlation between muscle mass and risk of carotid atherosclerosis suggests that preservation of muscle mass may modify atherosclerosis progression and future cardiovascular events [9,31].

### 3.2 Sarcopenia and peripheral arterial disease in patients with T2DM

Peripheral arterial disease (PAD) has been considered responsible for deterioration of muscle strength and function. Patients with PAD was shown to have weaker hip, knee, ankle, and plantar muscles and have slower gait compared to those without PAD [38]. Despite being primarily a vascular disease, all known etiological factors of sarcopenia are present in both PAD and T2DM, such as the presence of: (1) oxidative stress and inflammation; (2) skeletal muscle mitochondrial dysfunction; (3) inhibition of pathways regulating muscle synthesis or protection via mTOR, reperfusion injury salvage kinase (RISK), and survivor activating factor enhancement (SAFE) pathways; and (4) activation of molecules associated with muscle breakdown [38]. Reduced circulatory flow in PAD may result in ischemia, leading to poor muscle mass, lower muscle strength and reduced performance parameters. Pain associated with PAD also leads to reduced physical activity and exercise tolerance, contributing to poorer muscle health [39].

### 3.3 Sarcopenia and stroke in patients with T2DM

Patients with T2DM are at increased risk of both ischemic and hemorrhagic stroke. In their systematic review, Su et al. [40] reported that the estimated prevalence of stroke-related sarcopenia is 42%. Nevertheless, it is more difficult to make a full assessment of the exact contribution of T2DM-related complications in the prevalence of stroke-related sarcopenia as T2DM and stroke share overlapping risk factors and disease process. Stroke limits both physical activity and nutritional intake of old-aged T2DM patients [41]. Additionally, the state of systemic inflammation, including that derived from T2DM, was reported to be associated with sarcopenia and poorer functional outcome in the recovery stage of stroke [42]. It is not yet known whether sarcopenia increases the risk for stroke, but based on current data, it is reasonable to speculate that T2DM patients with stroke will have increased risk for sarcopenia due to physical inactivity and mobility limitation.

### 3.4 Sarcopenia and coronary arterial disease in patients with T2DM

It is known that T2DM and coronary artery disease (CAD) are linked. CAD is the main cause of death in T2DM with odds ratio of 4.56 (95% CI 3.53-5.89) compared to those without T2DM and CAD [32]. Different factors play roles in increasing the risk for atherosclerotic CAD in T2DM patients, such as the presence of hyperglycemia, dyslipidemia, insulin resistance, dysfunction of endothelial cell and vascular smooth muscle, impaired platelet function and coagulation abnormalities [43]. In contrast to the relationship between T2DM and CAD, a direct relationship between sarcopenia and CAD remains unclear. Kang et al. [10] investigated the relationship between sarcopenia and major cardiovascular events in CAD. They concluded that low skeletal muscle mass is a predictor of adverse clinical outcome, measured as all-cause mortality and major adverse cardiovascular event (MACE) in patients with CAD who underwent PCI [10]. The underlying mechanism of how sarcopenia might affect CAD seemed to be related to insulin resistance. Data from 13,644 adults showed that very low muscle mass is a risk factor for insulin resistance, and that higher muscle mass is associated with better insulin sensitivity because muscle is the main location of insulin-mediated glucose disposal [31]. Relative muscle mass was also negatively correlated with prevalence of coronary calcification in a study involving 31,108 asymptomatic adults without cancer, T2DM or known cardiovascular disease, further supporting the notion that low muscle mass is an independent risk factor for CAD [44]. Several factors that are thought to contribute to this are physical inactivity leading to low muscle mass, as well as levels of CRP, IL-6, and fibroblast growth factor 21 (FGF-21). High CRP level was associated with both the risk of losing muscle mass and risk of myocardial infarction and stroke. IL-6 was negatively associated with muscle mass but IL-6 receptor-related pathway was associated with CHD [45]. FGF-21 is a myokine in skeletal muscle induced by insulin stimulation that improve left ventricular dysfunction after MI in a murine model [44]. Taken together, these data show that although evidence are scarce, sarcopenia are related to CAD via mechanisms that involve inflammatory mediators and insulin resistance.

## 4 Relationship between sarcopenia and microvascular complication of diabetes

### 4.1 Sarcopenia and diabetic nephropathy

The prevalence of diabetic nephropathy, also known as diabetic kidney disease (DKD), is estimated around 24.4% to 40% that varies based on several factors such as age and clinical setting [46]. It is one of the most common etiologies for end-stage renal disease and strongly related with increased risk of mortality due to cardiovascular disease [47]. The pathophysiology of DKD begins with chronic hyperglycemia that stimulates humoral mediator, such as TGF-β, and growth factors causing structural kidney abnormalities such as deposition of extracellular matrix and impairment of basal glomerulus permeability [47].

The relationship between sarcopenia and nephropathy is speculated to be bidirectional. It is evident that chronic kidney disease (CKD) itself is a model of accelerated premature aging model which increases the risk of sarcopenia [48]. Muscle loss is often found in CKD patients, especially among advanced stage CKD and dialysis patients due to increased muscle degradation, decreased muscle synthesis, chronic inflammation, insulin resistance, and muscle fiber atrophy. On the other hand, albuminuria and decline in eGFR are closely related to insulin resistance, chronic inflammation, oxidative stress and endothelial dysfunction which also play a role in the pathophysiology of sarcopenia [49].

Several studies showed significant association between sarcopenia and albuminuria. Bouchi et al. [50] found significant association of sarcopenia and progression of albuminuria with HR 2.61 (95% CI = 1.08-6.31, p = .034). A systematic review analyzing the association between sarcopenia and renal function among T2DM patients, found increased risk of albuminuria among sarcopenia groups aged < 60 years old with OR = 2.38 (95% CI = 1.37, 4.15, I2 = 69%) [51]. An observational study by Pechmann et al. [52] found an increased risk of developing sarcopenia in the presence of albuminuria with OR = 2.84 (95% CI = 1.07-7.68, p = .04). Considering the close relationship between DKD and the progression of sarcopenia, even before elderly, there is a need to conduct further studies investigating the impact of sarcopenia and T2DM to kidney function in productive age population and earlier kidney damage marker.

### 4.2 Sarcopenia and diabetic neuropathy

The prevalence of neuropathy as the microvascular complication of T2DM could be as high as 61.8% with age, duration of T2DM, glycemic control, and other metabolic comorbidities as the risk factors [53]. In uncontrolled T2DM, damaged intraneural capillaries alter the blood supply to the peripheral nerves causing sensory loss, pain, and muscle weakness, which accelerates muscle mass loss. Diabetic neuropathy is strongly correlated with the incidence and progression of sarcopenia [54].

Previous observational studies have shown consistent higher prevalence of diabetic neuropathy assessed by both clinical scoring system and nerve conductive study among persons with sarcopenia compared to those without sarcopenia [55]. In the study by Yang et al. [55], diabetic neuropathy was more prevalent (p = .07) in T2DM patients with sarcopenia (80%) compared to T2DM patients without sarcopenia (70.3%). *Neuropathy Symptoms Score* (NSS) was independently associated with sarcopenia (OR = 1.507 [95% CI = 1.011-1.526, p = .039) [54]. Earlier evidence by Andersen et al. [13] in 2004 also found that diabetic neuropathy presented with neuropathy rank-sum score (NRSS) was negatively correlated with ankle strength (r = -0.45, p <. 01) and knee strength (*r* = -0.42, p < .02). In general, all of these studies analyzing the relationship between sarcopenia and diabetic neuropathy were conducted in older populations, which matched the common knowledge that both risk of neuropathy and sarcopenia increase with age. However, studies explaining how sarcopenia affects diabetic neuropathy are insufficient.

### 4.3 Sarcopenia and diabetic retinopathy

Diabetic retinopathy is one of the most common microvascular complications of T2DM which may lead to visual loss and blindness [56]. The prevalence of global diabetic retinopathy between 2015 to 2019 according to IDF atlas was approximately 27% with the Western Pacific Region as the highest prevalent area of diabetic retinopathy. The pathophysiology of diabetic retinopathy involves hyperglycemia-induced vascular damaged due to AGEs, chronic inflammation, activation of several pathways such as polyol and PKC, as well as neurological impairment that leads to pericyte loss [56]. Non-proliferative diabetic retinopathy (NPDR) in an early stage is characterized by increased vascular permeability, capillary occlusion, microaneurysms, hemorrhages, and hard exudates. In later stages, proliferative diabetic retinopathy (PDR) is marked by neovascularization [57].

Although diabetic retinopathy may not be directly associated with muscle weakness or muscle atrophy in sarcopenia, the presence of diabetic retinopathy increases the risk of fall (OR = 1.31; 95% CI = 1.07-1.60; p = .008) compared to persons without T2DM [58]. Those with diabetic retinopathy were found to have a high degree of restriction associated with mobility including public transport use, obstructed locomotion, and fear of falling [59]; mobility limitation may lead to the development of sarcopenia [40]. The relationship between sarcopenia and diabetic retinopathy also was investigated in a community-based study among T2DM elderly patients in Malaysia that found the prevalence of diabetic retinopathy in the T2DM group with sarcopenia was lower (28.8%) compared to the T2DM without sarcopenia group (71.2%) [60]. A previous observational study by Andersen et al. [13] also found no correlation between degree of diabetic retinopathy to ankle and knee muscle strength. However, opposite findings were found in a cross-sectional study by Fukuda et al. [11] in which the prevalence of sarcopenia was significantly increased with progression of diabetes retinopathy and PDR, but not NPDR, significantly increasing the risk of sarcopenia (OR = 7.78, 95% CI = 1.52-39.81, p = .014) and low hand grip strength (OR = 6.25, 95% CI = 1.15-33.96, p = .034). Hence, further investigation on the relationship between sarcopenia and diabetic retinopathy needs to be established.

## 5 Summary

T2DM and sarcopenia are closely related in bidirectional mechanism whereas the relationship of sarcopenia and chronic complications of diabetes still needs further investigation. T2DM may lead to sarcopenia mainly through insulin resistance, inflammation, oxidative stress, and AGEs. Whereas sarcopenia may affect T2DM through decreased production of myokines and through lipid accumulation in skeletal muscle that is associated with metabolic disturbance and inflammation. Together, T2DM and sarcopenia cause a dual health burden as both factors are associated with increased risk of fall and functional impairment, as well as macrovascular and microvascular complications. Therefore, early screening and diagnosis of sarcopenia among patients with diabetes, even at younger ages are essential. Comprehensive management of both T2DM and sarcopenia, emphasizing exercise and nutrition, needs to be elaborated in all patients to prevent the dual burden of T2DM and sarcopenia.

## Abbreviations

DM: Diabetes mellitus

GLUT: Glucose transporter

IL: Interleukin

TNF: Tumor necrosis factor

IRS: Insulin receptor substrate

HOMA-IR: Homeostatic model assessment for insulin resistance

JNK: c-Jun N-terminal kinase

NF_k_B: Nuclear factor kappa B

mTORC: Mechanistic target of rapamycin complex

AGE: Advanced glycation end-product

PAD: Peripheral arterial disease

CAD: Coronary artery disease

RAAS: Renin angiotensin aldosterone system

DKD: Diabetic kidney disease

PDR: Proliferative diabetic retinopathy

NPDR: Non-proliferative diabetic retinopathy
